# The influence of excessive stress on medical students in the Czech Republic – national sample

**DOI:** 10.1186/s12909-023-04157-9

**Published:** 2023-03-17

**Authors:** M. Palička, M. Rybář, B. Mechúrová, N. Paličková, T. Sobelová, K. Pokorná, J. Cvek

**Affiliations:** 1 University Hospital Ostrava, Ostrava, Czech Republic; 2 Czech Technical University, Prague, Czech Republic; 3 Charles University, Prague, Czech Republic; 4 University of Ostrava, Ostrava, Czech Republic

**Keywords:** Excessive stress, Medical students, Study dropout, Alcohol abuse, Medicaments abuse, Somatic problems

## Abstract

**Purpose:**

The Czech Republic has been dealing with a long-term shortage of doctors, which, according to demographic forecasts, will continue to worsen due to the retirement of stronger generations of doctors in contrast to the gradual aging of the population, which will require more health care over time. The country´s political set is trying to respond to this shortage and demographic forecasts by gradually increasing financial funding of medical faculties with the aim of increasing the number of graduates of the program in the field of general medicine.

**Methods:**

Anonymous questionnaire survey was conducted among students and graduates of general medicine at all eight Czech medical faculties. A total of 3183 respondents participated in the survey. There were 2843 medical students, which represents approximately 28% of all medical students in the Czech Republic. The distribution of respondents within the study years was approximately even and approximately corresponded to the real distribution of students between individual faculties in country, which makes survey a national sample. The statistical processing was performed in the statistical software R. Apart from the basic comparison using percentage relative frequencies and Pearson´s chi-squared test, in this study we used Odds ratios (OR) with CI 0,95 from logistic regression model for a better interpretation of some outputs.

**Results:**

The results show that the vast majority of Czech medical students experience excessive stress during their studies, which increases the risk of students´ somatic problems (OR = 4.89, CI 0.95 = (4.11;5.83), *p* < 0.001)., targeted alcohol use (OR = 2.29, CI 0.95 = (1.73;3.04), *p* < 0,001) and the use of anxiolytic or antidepressant medication to reduce it (OR = 2.99, CI 0.95 = (2.24;4.01), *p* < 0.001). Students experiencing higher levels of excessive stress are more likely to leave their studies based on their own decision (4.20 (CI 0.95 (3.39;5.19), *p* < 0.001) and not to enter clinical practice after graduation (OR = 2.62, CI 0.95 = (2.06;3.33), *p* < 0.001).

**Conclusions:**

The survey shows the need for an open discussion at the highest level about the possibilities of reasonable reduction of unnecessary stress during medical studies. Medical students in the Czech Republic are exposed to excessive stress with all the consequences described above. All that remains is to state the existence of unnecessary components of stress, which represent an opportunity to reduce it, thereby achieving better conditions for studying, improvement in the staff situation in the Czech healthcare system and a reduction in inefficiently spent financial resources for the education of young doctors.

**Trial registration:**

No registration.

**Supplementary Information:**

The online version contains supplementary material available at 10.1186/s12909-023-04157-9.

## Introduction

General medicine is one of the most sought-after and prestigious university fields in the Czech Republic. Tens of thousands of applicants apply to the eight medical faculties every year. The study itself is considered one of the most demanding across disciplines, both for its length and the amount of required knowledge and skills, as well as for its psychological demands, competitive environment and encounter with human suffering. All of the above factors and circumstances can cause excessive stress in medical students [1].

One of the many definitions defines stress as a subjectively felt state of imbalance between the demands on individual and the capacities to cover these demands [2, 3]. In the case of medical students, these are requirements resulting from the curricula of individual medical faculties, which put high demands on their students [4].

Exposure to long-term and excessive stress leads to both psychological (fear, anxiety, panic attacks, depressions, memory disorders), and somatic problems (vomiting, diarrhea, rash, insomnia, weight loss, weakness, shortness of breath, palpations) [5–7]. Students suffering from excessive stress may more often decide to consume alcohol or medications (anxiolytics and antidepressants) [1]. All of the above mentioned consequences lead to a decrease in the quality of life of students. Exposing students to excessive levels of stress can significantly increase the tendency of students dropping out. Last but not least, it is necessary to note, that students who successfully complete their studies bear the consequences of excessive long-term stress into their professional and personal lives.

The Czech Republic has been dealing with a long-term shortage of doctors, which, according to demographic forecasts, will continue to worsen due to the retirement of stronger generations of doctors in contrast to the gradual aging of the population, which will require more health care over time. The country´s political set is trying to respond to this shortage and demographic forecasts by gradually increasing financial funding of medical faculties with the aim of increasing the number of graduates of the program in the field of general medicine.

The main goal of the survey was to objectify the problematics of subjective perception of excessive stress by students of general medicine in the Czech Republic and to assess the effect of this stress on their life, their motivation to further remain in the system of Czech healthcare and possibly to identify the relationship between excessive stress and failing to complete medical studies. The partial goal of our work was to map the sources of stress of medical students and to try to name the unnecessary components of overall stress, the systematic reduction of which can contribute to improving the conditions of study and the quality of life of medical students while preserving the professional level of graduates of Czech medical faculties.

## Methods

**Fig. 1:**
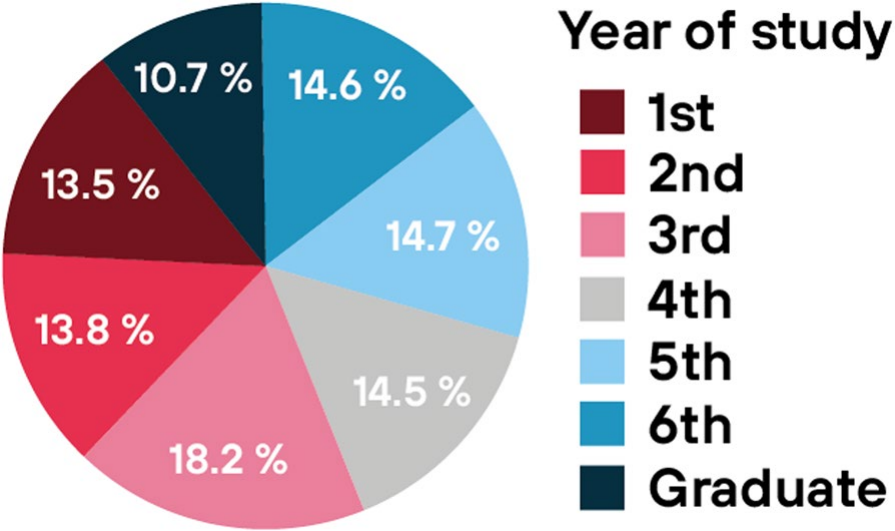
Year of study

**Fig. 2:**
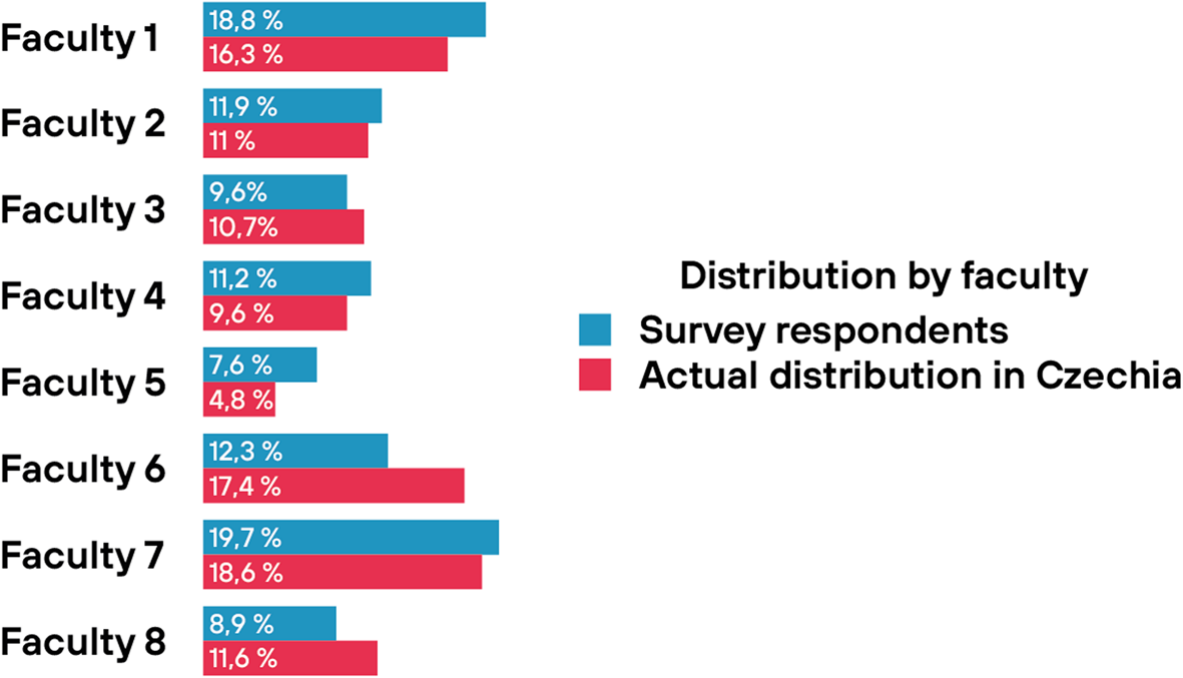
Distribution by faculty

Anonymous questionnaire survey was conducted among students and graduates of general medicine at all eight Czech medical faculties. A total of 3183 respondents participated in our survey. There were 2843 medical students, which represent approximately 28% of all medical students in the Czech Republic (see annual reports of individual medical faculties). The rest of the respondents (340) were graduates within five years of graduation. Nearly 73% of this survey were women. The distribution of respondents within the study years was approximately even (Fig. 1). The distribution of respondents in our survey approximately corresponded to the real distribution of students between individual faculties in the Czech Republic, which makes our survey a national sample (Fig. 2).

Questionnaire survey “PM 2021” contained a total of 22 questions, of which 19 questions were closed-ended and 3 questions were open-ended (Supplementary material 1 and 2). The closed-ended questions at the beginning of the questionnaire related to basic demographic data about the respondents (gender, year and studied faculty), other questions contained statements with a range of possible answers: 1-strongly agree, 2-moderately agree, 3-undecided, 4-moderately disagree, and 5-strongly disagree. The open-ended questions were aimed at mapping specific sources of stress during the course study and the motivation for continuation of stay in the Czech healthcare system. Open-ended questions about specific sources of stress were divided into six categories that are often used in works with a similar focus [8, 9]: academic-related stressors (ARS), interpersonal- and intrapersonal-related stressors (IRS), teaching- and learning-related stressors (TLRS), social-related stressors (SRS), drive- and desire-related stressors (DRS), group activity-related stressors (GARS). In case students reported more than one specific source of stress, the first two responses were included in the results. A total of 2505 respondents answered this open question. For the subsequent categorization of the data, we included only those students who answered the question. Categorization was performed by one researcher. Responses to specific stress factors were then categorized for quantitative graphical processing. Answers to all questions in our survey were optional for respondents.

Collection of data took place in November and December 2021 through online questionnaire, which respondents from the target group were invited to fill out. The data was collected and stored via the professional online platform survio.com, which with its security meets all the regulations of the regulatory authorities in the Czech Republic and the European Union (GDPR, ISO, OV SSL). The data collection took place in cooperation with the study departments of the individual medical faculties, which sent a total of three invitations to fill in the questionnaire to the students by e-mail. The first call was sent in the first week of November 2021, and the next two reminders each 28 days after the previous call.

The statistical processing was performed in the statistical software R (R Core Team, 2022). *P*-values < 0.05 will be considered statistically significant. Apart from the basic comparison using percentage relative frequencies and Pearson´s chi-squared test, in this study we used Odds ratios (OR) with CI 0,95 from logistic regression model for a better interpretation of some outputs.

The questionnaire was anonymous in its entirety and did not collect any personal data of the respondents. According to the laws of the Czech Republic, a questionnaire survey does not require the approval of the ethics commission. All methods were carried out in accordance with relevant guidelines and regulations. The need for informed consent was deemed unnecessary according to national regulations as assessed by the Ethics Committee of the Faculty of Medicine, University of Ostrava.

## Results

### Overall level of perceived stress

For the purposes of our survey, we defined stress as follows: The organism's natural and integral response to stimuli and situations of the external and internal environment, which are subjectively perceived by the organism as demanding, burdensome or dangerous. A total of 94.8% of all respondents agreed with the statement “I experience excessive stress while studying medicine “, of which 64.36% of respondents chose the answer “strongly agree “ and 30.44% of all respondents chose the answer “moderately agree”.

**Fig. 3:**
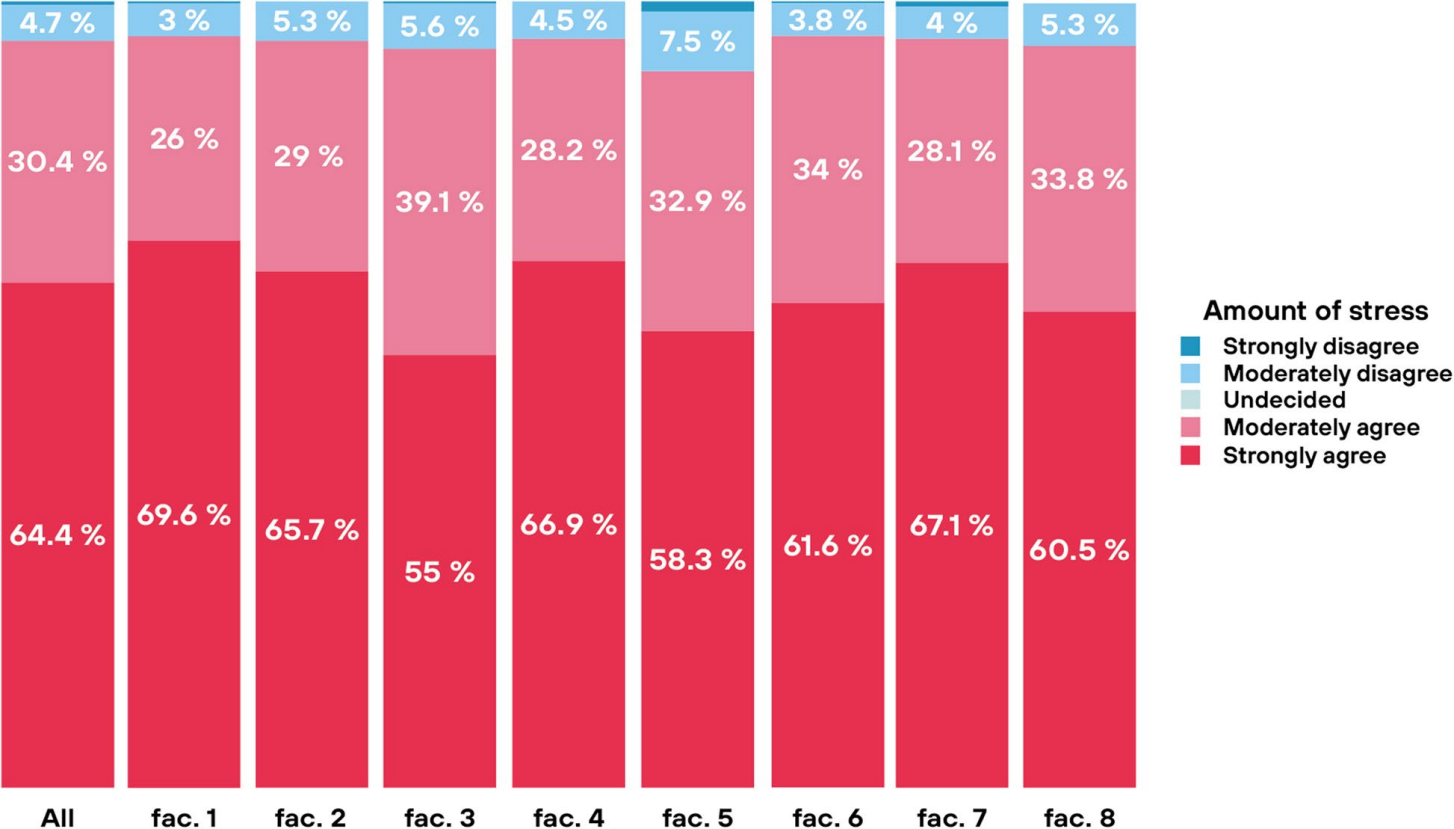
Amount of stress among faculties

Figure 3 shows that students report a comparable overall level of stress at individual faculties. There are certain statistical differences between the faculties, but they are not significant from the point of view of the monitored issue. During the data analysis, we did not find any statistically significant differences in the perception of stress among students across individual years of study. Similarly, we did not find a difference between current students and graduates included in the survey.

### The consequences of excessive stress on Czech students of medicine

According to the questionnaire survey, 40.3% of all respondents (1 – strongly agree) experienced somatic problems related to stress during their studies. Students who answered that they experience stress during their studies (1 – strongly agree) said that they experience somatic problems (1 – strongly agree) in 52.60%. On the other hand, only 18.05% of students who did not suffer from stress (5 – strongly disagree, 4 – moderately disagree, 3 – undecided, 2 – moderately agree) said that they experienced somatic problems during their studies. Students who reported a higher level of stress during their studies experience somatic problems more often than students reporting a lower level of stress (OR = 4.89, CI 0.95 = (4.11;5.83), *p* < 0.001).

The goal of our survey was also the analysis of abuse of alcohol and pharmaceuticals for the targeted reduction of study-related stress. 12.19% of all respondents strongly agreed with the statement about the targeted consumption of alcohol to reduce stress. Overall, 14.84% of respondents strongly agreed with the statement about the use of anxiolytic or antidepressant medication. Excessive stress (1 – strongly agree) experienced during medical studies increases the risk of targeted use of alcohol (OR = 2.29, CI 0.95 = (1.73;3.04), *p* < 0,001) and medication to reduce this stress (OR = 2.99, CI 0.95 = (2.24;4.01), *p* < 0.001).

Students who answered that they experience stress during their studies (1 – strongly agree) said that they wanted to leave their medical studies (1 – strongly agree) in 33.40%. On the contrary, students who did not suffer from stress (5 – strongly disagree, 4 – moderately disagree, 2 – moderately agree) stated that they wanted to leave their studies only in 10.65%. According to OR = 4.20 (CI 0.95 = (3.39;5.19), *p* < 0.001), students experiencing a higher level of stress are more prone to drop out of their studies based on their decision than students who experience a lower level of stress during their studies.

Students who answered that they experience stress during their studies (1 – strongly agree) said that they considered not entering clinical practice (1 – strongly agree) in 19.15%. On the other hand, only 8.45% of students who did not suffer from stress (5 – strongly disagree, 4 – moderately disagree, 3 – undecided, 2 – moderately agree) said that they thought about not entering practice. Students who experience higher levels of stress may be less likely to enter clinical practice after graduation than students experiencing lower levels of stress during their studies (OR = 2.62, (CI 0.95 = (2.06; 3.33), *p* < 0.001).

In situations where psychological problems exceed the tolerable limit, the ability to seek professional help is important in order to prevent serious health consequences, such as the burnout syndrome, severe depression or suicide attempts. 18.4% of all respondents to our survey strongly agreed with the statement about seeking professional help in connection with excessive stress (1 – strongly agree) during studies. Excessive stress experienced during medical studies increases seeking professional help (OR = 4.79, CI 0.95 = (3.50; 6.54), *p* < 0.001).

To illustrate the complexity of the point of view, 75% of all respondents agreed with the statement that on average they spend more than 8 h a day studying medicine. The majority of respondents also agreed with the statement that they do not have enough time for leisure activities (71%) and their loved ones (64%).

### The main sources of stress

**Fig. 4:**
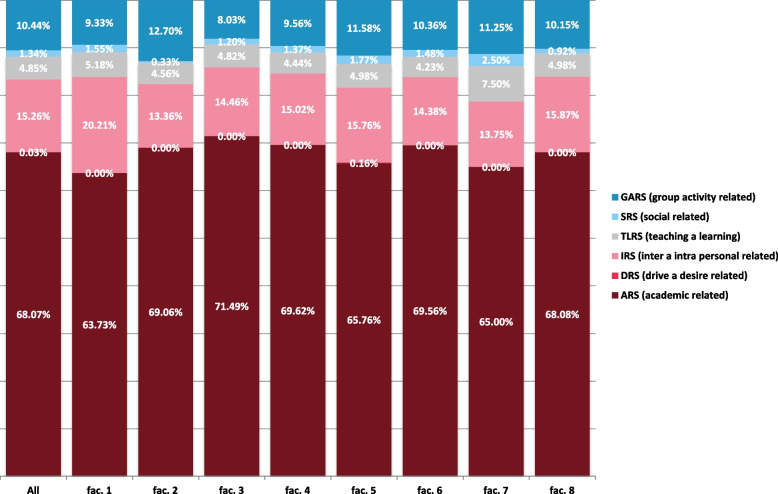
Source of stress

When searching for specific factors causing stress in medical students, we asked the students an open-ended question about specific situations causing stress in them during their studies. We present the results in categorized form (Fig. 4).

When asked about specific factors causing stress, the prevailing answers were from ARS category (exams, falling behind in reading schedule, the amount of knowledge required, lack of time for friends, family and leisure activities), which were given by a total of 68.07% of respondents. The second most frequently mentioned cause of stress was IRS category (verba/physical/abuse by teachers or other students and other conflicts) which was given by a total of 15.26% of respondents. GARS category (fear of being unprepared for the work of a doctor, need to do well (imposed by others), participation in class presentation) was given by a total of 10.44% of respondents. The SRS (unable to answer questions from patients, facing illness or death of the patients, talking to patients about personal problems), TLRS (not enough feedback from teachers, lack of recognition for work done), and DRS (unwillingness to study medicine, parental wish for you to study medicine) categories were each mentioned in less than 5% of cases (Fig. 4).

All the above-mentioned stressors vary with variability across individual medical faculties, the differences between faculties are are statistically significant (Chi -square test; *p* < 0.001), which means an opportunity for faculty managements to name and reduce unnecessary stress with specific measures. Despite the aforementioned variability, all faculties in the Czech Republic show a comparable overall level of reported stress (Figs. 3 and 4).

### Do women and men perceive excessive stress during studies in the same way?

An indisputable trend in recent years at Czech medical faculties is the growth of predominance of women, which also corresponds to the distribution of respondents in our survey.

When comparing the subjective experience of stress between genders, women report highest stress level (1 – strongly agree) more often than men (OR = 1.92, CI 0.95 = (1.64; 2.26), *p* < 0.001). Similar result was obtained when comparing reported somatic problems (OR = 1.71, CI 0.95 = (1.45;2.03), *p* < 0.001), probability of dropping out (OR = 1.24, CI 0.95 = (1.03;1.49), *p* < 0.001) and when comparing the frequency of seeking professional help (OR = 1.91, CI 0.95 = (1.46;2.48), *p* < 0.001).

When comparing the use of anxiolytics/antidepressants, we get OR = 1.67, (CI 0.95 = (1.27; 2.19), *p* < 0.001). Only the use of alcohol in connection with stress is reported more often by men (OR = 0.65, CI 0.95 = (0.51; 0.83), *p* < 0.001).

## Discussion

Our survey provides a comprehensive, and in the Czech context, unique view of the stress subjectively felt by students of general medicine throughout the country (Czech Republic), its effect on them and their motivation to remain in the Czech healthcare system. Thanks to the scale and the even distribution of students across the faculties, we were able to name the main sources of stress at individual medical faculties.

Our above-presented results show that the vast majority of Czech medical students experience excessive stress during their studies, which increases the risk of students´ somatic problems, targeted alcohol use and the use of anxiolytic or antidepressant medication to reduce it, which affects their lives. Students experiencing higher levels of excessive stress are more likely to leave their studies based on their own decision and not to enter clinical practice after graduation.

In some parts, the work of the Thai authors Pitanupong et al., who investigated the proportion of medical students who considered leaving medicine during their studies, and tried to identify the reasons for these considerations, is comparable to our survey. Their results show that 22.9% of students admitted to considering leaving medicine in the preclinical section, while 22.6% of students admitted to leaving medicine in the clinical section [10]. As the main reasons for these considerations, students cited the difficulty of studying, dissatisfaction with the study environment and lack of interest in the content of the lesson. Compared to these results, we note that Czech medical students admit that they think about leaving medicine more often, a total of 30.4% (1 – strongly agree). Given the higher reported level of stress in women, this difference can be partly explained by the proportion of women in our survey, where women represented 54% in the above-cited Thai study and 73% in our survey. However, this effect will only be slight.

Excessive stress and anxiety in medical students are associated in the literature with excessive alcohol consumption with a prevalence of around 20%. This prevalence is higher than in the non-medical peer group, despite relatively high alcohol consumption in the control (non-medical) population [11–18]. In our survey, the prevalence of alcohol consumption reaches almost 30% (1 – strongly agree, 2 – moderately agree), for men up to 35%. The statement in the questionnaire that the respondents agreed with was defined as the use of alcohol for the purposeful reduction of excessive stress (i.e. not for fun or during celebrations or other occasions).

In accordance with our results, high demands on students are repeatedly mentioned in terms of study volume, competitive environment among medical students, lack of time for friends, family and leisure activities, high demands of family and society, or great responsibility of the future profession. literature as the main and specific sources of stress. Others include frequent investigations, worries about the future, loneliness or encounters with death [19, 20]. A 2018 survey of more than 1,100 students by American authors [21] adds other factors such as faculty teaching systems, teacher attitudes, and student-hostile environments.

Nowreen and Ahad's work focusing on sources of stress in first year medical students of Sher-i-Kashmir Institute of Medical Sciences in Pakistan indicates that ARS (63.95%) is the most common source of severe stress among students, followed by TLRS (43.02%), IRS (32.55%), DRS (25.58%), SRS (19.76%), and GARS (15.11%) [8]. The work of Melak and the collective indicates in their work as a source of strong stress ARS (31.5%), followed by IRS (30.4%), TLRS (28.1%), SRS (21.2%), GARS (16.9%), and DRS (13.8%) [9].

Based on our work, the most common source of stress among students is ARS (68.07%), followed by IRS (15.26%), GARS (10.44%), TLRS (4.85%) and SRS (1.34%). However, this comparison is imprecise due to the different methodology of our work and further work will be needed to verify the results.

In literature, the female gender is repeatedly associated with a higher risk of feeling excessive stress, but also of burnout syndrome [22, 23]. The lifetime risk of developing depression is higher in women than men in the general population [15, 24, 25]. On the other hand, several studies can be found that find no difference in the prevalence of depression among medical students by gender [15, 26–33]. Even among our respondents, women reported highest stress level (1 – strongly agree) more often than men (OR = 1.92, CI 0.95 = (1.64; 2.26), *p *< 0.001). Similarly, when comparing reported somatic problems, women are more often burdened (OR = 1.71, CI 0.95 = (1.45; 2.03), *p* < 0.001) and likewise when comparing the frequency of seeking professional help (OR = 1.91, CI 0.95 = (1.46;2.48), *p* < 0.001). When comparing the use of anxiolytics/antidepressants, we get a result OR = 1.67, CI 0.95 = (1.27; 2.19), *p* < 0.001. Only the use of alcohol in connection with stress is reported more often by men (OR = 0.65, CI 0.95 = (0.51; 0.83), *p *< 0.001). The question in this area and in the context above is that to what extent our results reflect the general tendency of women to experience life situations as stressful and to what extent our results are a reflection of hostility, discrimination or sexism at Czech medical faculties.

Works published so far with a similar focus indicate different percentages of students experiencing excessive stress during their medical studies. Work by Konjengbam et al. indicates a prevalence of stress of 28.4% among medical students studying in India [34]. Other results were obtained by Fares et al. with a cohort of Lebanese medical students, who in their studies found excessive stress in 62% of medical students [23]. The work of A.N. Supe talks about the prevalence of stress of 73% among medics in Seth G.S. Medical College in India with higher prevalence in higher grades [35]. A recently published meta-analysis by Pu Peng and colleagues reports excessive stress in 34% of medical students during the covid-19 pandemic (CI 0.95 = (27%; 42%) [33]. Compared to these works, 64.36% of Czech medical students report high level of excessive stress (1 – strongly agree) with all its consequences, which we described above. In this case, it must be said that the works cited had different methodologies and further research will be needed to clarify the claims.

### Why do we need to reduce excessive stress?

Based on what we mentioned above, it can be said that Czech medical students are exposed to excessive stress with all its consequences during their studies. Psychological and somatic problems or the use of alcohol and pharmaceuticals to reduce stress are not desirable phenomena for future doctors. Although studying medicine is demanding in itself and requires a great deal of energy, time, patience and sacrifice, in our survey we have shown that there is unnecessary stress, which represents up to tens of percent across faculties. The degree of representation of individual stressors varies between different faculties. It is necessary to strive for a systematic reduction of unnecessary stress across the faculties by means of targeted measures, which can improve conditions for studying without the unnecessary consequences described above.

In addition to reducing the above-mentioned consequences of excessive stress, it is also necessary to consider the systemic and economic side of the matter. The society invests high costs in the education of one students, which will potentially be wasted if such a student does not finish their studies due to excessive stress or pursues another field after graduation. Reducing unnecessary stress during studies by both medical faculties and students can undoubtedly save the entire system considerable financial resources and perhaps even improve the staff situation in the Czech healthcare sector.

### Strengths and limitations of study

Since the preparation of the survey, we have been aware of a possible sampling bias, given that students who are more sensitive to stress may be more likely to fill out a similarly focused questionnaire, but due to the scope of our survey and the equal representation of students from individual faculties, our survey provides a robust result. Additionally, similar cohorts of students within faculties were compared.

Our survey took place during the coronavirus pandemic, when some medical students were ordered to help the overburdened healthcare system. At that time, the majority of teaching at Czech universities took place via distance learning, and it was medical students in the upper years who were exempted from the regulation, when practical teaching was mostly preserved. This bias must also be taken into account when evaluating our results.

The weakness of our work is the methodology of our questionnaire, which is not comparable to other previously published works where standardized questionnaires (GHQ-12, DASS) were used. The composition of the questionnaire was consulted with the management of individual medical faculties. The questionnaire was compiled with the aim of obtaining as many specific answers as possible, which were subsequently provided to individual faculties with the aim of making relevant changes. In this regard, our results are hypothesis-generating and require follow-up surveys and in-depth analysis.

## Conclusions

Our work shows the need for an open discussion at the highest level about the possibilities of reasonable reduction of unnecessary stress during medical studies. Medical students in the Czech Republic are exposed to excessive stress with all the consequences described above. All that remains is to state the existence of unnecessary components of stress, which represent an opportunity to reduce it, thereby achieving better conditions for studying, improvement in the staff situation in the Czech healthcare system and a reduction in inefficiently spent financial resources for the education of young doctors.

### What can medical faculties do?

We see an opportunity for change in, among other things, the transparent, reproducible and fair verification of students´ knowledge, the specific stating of the required range of knowledge, and above all the clarity of the criteria for successful completion of the course. We can draw inspiration from the verified experiences of foreign universities. We also perceive the need for education on the topic of mental illness in medicine and education of students in the basics of psychohygiene, effective learning methods and time management. Education and support in this issue should be considered as one of the priorities of the education of current and future doctors, as it has the potential to improve the overall quality of life and therefore the overall level of services in the current healthcare sector. Every student should have access to contacts for seeking professional help. It is the responsibility of medical schools to increase awareness among students about where they can find these contacts if needed (Table 1).

**Table 1:** Options for solving the causes of the main stress groups by medical faculties and students

	**Faculties´ options**	**Students´ options**
**Stress from exams (volume of study, implementation of exams)**	- Fairness, reliability and predictability of examining - Clear framing of the required range of knowledge	- Evaluation of teaching quality- Honest and systematic preparation for exams - Using the principles of effective time management - Using effective study methods
**Stress from the medical environment and pressure**	- Education and enlightment of students - Providing contacts for professional advice - Active search for factors of unnecessary stress	- Using the principles of psychohygiene - At the right time seeking help in cases where the stress exceeds the bearable limit

### What can medical students do?

We can only recommend honest and systematic preparation for the exams to students, without which it is difficult to cope with the demanding study of medicine. We also consider the regular evaluation of teaching quality, which is a standard part of the academic year at all Czech medical faculties, to be very important. To reduce stress during studies, it is also recommended to use the principles of proper time management and effective learning methods. Last but not least, we would like to mention the observance of well-known principles of psychohygiene. In cases where stress during the course of study exceeds the tolerable limit, it is necessary to be able to seek professional help (Table 1).

## Supplementary Information

**Additional file 1: Attachment 1.** Questionnaire PM 2021.

**Additional file 2: Attachment 2.** Categorization of open-ended answers in question number 8 of the PM 2021 questionnaire.

## Data Availability

The datasets used and/or analysed during the current study available from the corresponding author on reasonable request.
