# Supplementary material for: The influence of excessive stress on medical students in the Czech Republic – national sample
Source: BMC Med Educ. 2023 Mar 17;23:168. doi: 10.1186/s12909-023-04157-9 (PMC10021045; doi:10.1186/s12909-023-04157-9)
Supplement: Supplementary file 1 — Additional file 1: Attachment 1. Questionnaire PM 2021. [file 12909_2023_4157_MOESM1_ESM.docx]

1. Gender
2. Year of study
3. Faculty
4. After completing my studies, I would like to devote myself to the field of study: (open-ended)
5. I consider myself an anxious person.
6. I experience excessive stress while studying medicine.
7. Studying medicine bring me more stress than any other activity in my life.
8. If you answered positively to the previous questions (very much or rather agree), please try to name specific factors or situations that cause you the most stress during your studies. (open-ended)
9. I feel that I do not have enough time left for leisure activities.
10. I feel like I don´t have enough time left for my loved ones.
11. I feel that other university study programs (in the medical school and in other faculties) are not as stressful as medicine.
12. In my free time, I keep thinking that “I should study“.
13. Friends and family often tell me that I study a lot.
14. I spend an average of more than 8 hours a day on medicine (compulsory education, studies, internships and other related activities).
15. I also experience study stress outside of exam periods.
16. During my medical studies, I experiences physical problems related to stress.
17. During my medical studies, I sought professional help to deal with study-related stress.
18. During my medical studies, I used anxiolytic/antidepressant medication.
19. During my medical studies, I purposefully used alcohol or other addictive substances to relieve stress.
20. During my medical studies, I wanted to leave my studies.
21. During my medical studies, I considered the possibility of not pursuing clinical practice in the healthcare sector after graduation.
22. If you have answered positively in the previous questions, please indicate other reasons that, apart from study stress, led to these considerations. (open-ended)

**Attachment 1: Questionnaire PM 2021**
