# Supplementary material for: The influence of excessive stress on medical students in the Czech Republic – national sample
Source: BMC Med Educ. 2023 Mar 17;23:168. doi: 10.1186/s12909-023-04157-9 (PMC10021045; doi:10.1186/s12909-023-04157-9)
Supplement: Supplementary file 2 — Additional file 2: Attachment 2. Categorization of open-ended answers in question number 8 of the PM 2021 questionnaire. [file 12909_2023_4157_MOESM2_ESM.docx]

1. Exams, credits, amount of subject matter
2. Psychological factors (fear of failure, perfectionism, pressure to perform, comparison with others, pressure from the environment, etc.)
3. Lack of free time
4. System factors, study organization
5. The approach of teachers and examiners
6. Unpreparedness for practice, responsibility, communication with patients
7. Health or psychological difficulties
8. Lack of time, amount of material to study in a short period of time, feeling that I am not keeping up
9. Other

**Attachment 2: Categorization of open-ended answers in question number 8 of the PM 2021 questionnaire**
